# Exposure to DDT and HCH congeners and associated potential health risks through khat (*Catha edulis*) consumption among adults in South Wollo, Ethiopia

**DOI:** 10.1007/s10653-021-00846-w

**Published:** 2021-02-17

**Authors:** Desta Woldetsadik, Marcel Pierre Simon, Dennis Knuth, Hillette Hailu, Araya Gebresilassie, Asmare Dejen, Rolf-Alexander Düring

**Affiliations:** 1 Department of Soil and Water Resources Management, Wollo University, Dessie, Ethiopia; 2 Department of Soil Science and Soil Conservation, Research Centre for BioSystems, Land Use and Nutrition (iFZ), Justus Liebig University Giessen, Giessen, Germany; 3 Department of Zoological Sciences, Addis Ababa University, Addis Ababa, Ethiopia; 4 Department of Plant Science, Wollo University, Dessie, Ethiopia

**Keywords:** Khat, Ethiopia, Organochlorine pesticides, Dichlorodiphenyltrichloroethane, Hexachlorocyclohexane, Gas chromatography–mass spectrometry, Solid phase microextraction

## Abstract

Khat (*Catha edulis*) chewing is widespread in the region of East Africa. Even low levels of organochlorine pesticides (OCPs) in khat could induce public health concern. In a market-based study, from five popular khat varieties, a total of 35 composite khat samples were analyzed for dichlorodiphenyltrichloroethane (DDT) and its main transformation products, and four hexachlorocyclohexane (HCH) isomers. Extraction was carried out by quick, easy, cheap, effective, rugged and safe method (QuEChERS). OCP concentrations were determined by head space solid phase microextraction coupled to gas chromatography-mass spectrometry (HS-SPME–GC–MS). Every sample contained β-HCH above the maximum residue limit set by the European Commission. For total DDT, this was the case for 25.7% of the samples. The ratios of (*p*,*p*′-DDD + *p*,*p*′-DDE) to *p*,*p*′-DDT were less than one for 85% of khat samples, demonstrating recent use of DDT in khat farmlands. Conversely, the ratio of β-HCH to total HCH varied from 0.56 to 0.96, implying historical input of technical HCH. Assuming a daily chewable portion of 100 g, dietary intakes of *p*,*p*′-DDT, total DDT and total HCH by adults ranged from 3.12 to 57.9, 6.49 to 80.2 and 39.2 to 51.9 ng (kg body weight)^−1^ day^−1^, respectively. These levels are below acceptable levels suggested by international organizations. Chewing khat showed lower non-cancer health risk, but showed relatively higher cancer risk in terms of OCPs. Because khat is chewed without being subjected to any treatment, uncertainties associated with estimated intakes and health risks should be low. Therefore, this practice is of great concern.

**Supplementary Information:**

The online version contains supplementary material available at (10.1007/s10653-021-00846-w).

## Introduction

Khat is an evergreen shrub that mainly grows in East Africa and the Arabian Peninsula (Al-Hebshi and Skaug 2005). It has been used for centuries as a mild stimulant. For most people, chewing khat is a mode of increasing energy and elevating mood (Badedi et al. 2020). The psycho-stimulating effect of khat is due to the alkaloid cathinone, which has a similar chemical structure as amphetamine (Lim et al. 2019). Although khat is one of Ethiopia’s largest crop by area of cultivation and the country’s second largest export earner, it is considered an illicit drug crop in an increasing number of countries (Shephard 2012; Cochrane and O’Regan 2016; Carrier and Klantsching 2018). On the other hand, the practice of chewing khat extends from East Africa and the Arabian Peninsula to Western countries such as Germany, the Netherlands and the UK (Gebissa 2010; Thomas and Williams 2013; Bongard et al. 2015; El-Menyar et al. 2015; Nabben and Korf 2017; Nordgren 2018). Although there is no comprehensive survey on the number of people who adopted the practice of chewing khat in Amhara Regional State, more than 27.3% of Ethiopia's men aged between 15 and 49 years are presumably involved in this practice (CSA 2012; Wondemagegn et al. 2017). In the past two decades, new patterns of khat use including morning chewing sessions have become very common in East Africa (Warfa et al. 2007; Beckerleg 2010; Ahmed et al. 2020). This has contributed to the increase in the amount and frequency of khat intake among users. In South Wollo, khat chewing is a very prevalent socio-cultural and recreational activity and the number of people who have adopted such practice is expected to be far above the national average (Estifanos et al. 2016). With growing concerns about food safety, attention needs to be focused on khat as it is an important part of the daily consumption pattern across South Wollo. Most of the khat sold in towns of the sub-region originates from the khat cultivation belts of eastern and western Amhara, Amhara Regional State, Ethiopia. Khat production is an important income source for a large number of farmers in the region (Cafer 2016; Cafer and Rikoon 2017; Ruder 2018).

Pesticides have been widely used to control pests and improve crop yield and quality (Delcour et al. 2015). Organochlorine pesticides (OCP) including the insecticides DDT (dichlorodiphenyltrichloroethane) and HCH (hexachlorocyclohexane) exhibit competitive advantages for crop production and productivity. However, owing to their toxicity, persistent nature, high biomagnification potential, and detrimental health effects to humans, DDT and HCH have been banned for agricultural purposes since the 1980s (Feng et al. 2003; Haylamicheal and Dalvie 2009) and are listed as persistent organic pollutants (POPs) in the Stockholm Convention. Ethiopia was also a signatory of this convention. Organochlorine pesticides generally increase the production of free radicals in the human body, and on the other hand decrease the activity of anti-oxidant enzymes, which leads to oxidative stress (Kumar et al. 2010; Araki et al. 2018). A recent case control study demonstrated the alterations in oxidative capacity among members of farmworkers exposed to a mixture of OCPs in Iran (Abbasi-Jorjandi et al. 2020). Indeed, the presence of *p*,*p*′-DDT in the serum of the study participants indicated the possible recent contamination of food crops with DDT. Also, a growing number of cohort and epidemiological studies linked OCPs with type 2 diabetes (Al-othman et al. 2015; Singh and Chan 2017; Daniels et al. 2018). For example, Cox et al. (2007) found that serum *p*,*p*′-DDT was significantly associated with self-reported type 2 diabetes among Mexican Americans after correcting for total serum lipids. Recently, Han et al. (2020) also revealed that individuals exposed to OCPs had the highest risk of developing type 2 diabetes after adjusting for known risk factors. Similarly, OCPs can also influence the immune defense, depending on factors such as dose and route of exposure, thus potentially increase susceptibility to develop COVID-19 (Tsatsakis et al. 2020). For example, in Northern Italy whereby mortality rate from COVID-19 is far higher than reported rates in other territories across Europe and beyond, 65% of water samples from rivers and lakes exhibited high levels of pesticides including *p*,*p*′-DDE and *p*,*p*′-DDT (Bornstein et al. 2020). While this might be a mere coincidence, a causality is possible and should be further investigated.

Due to improper storage conditions of huge amounts of obsolete pesticides, Ethiopia is considered to be one of the worst contaminated countries in Africa (Hussen et al. 2006; Haylamicheal and Dalvie 2009). In 2006, the WHO declared that it would support the use of DDT for malaria control. As a result, some countries including Ethiopia reconsidered its use (Eskenazi et al. 2008; Daba et al. 2011). This has created an opportunity for farmers to easily access OCPs for illegal use in farms from depots meant for vector control. Hence, DDT is still widely applied to ensure the safety of agricultural products and to control the transmission of vector borne-diseases such as malaria, dengue, leishmaniases, and trypanosomiasis (Hussen et al. 2006; Yohannes et al. 2014; Mekonen et al. 2017). The khat cultivation system in most parts of the country is characterized by the use of OCPs (Mohammed 2010; Daba et al. 2011; Serda et al. 2015; Mekonen et al. 2017; Ruder 2018). Descriptive analysis of DDT application in different khat-based agroecosystems illustrated the complexity of the usage patterns. To make the chewable parts of khat visually enticing (i.e. glossy), DDT is applied even during the growing season (Mohammed 2010; El-Zaemey et al. 2015; Mekonen et al. 2017; Regassa et al. 2020). Cognizant of this trend, *p*,*p*′*-*DDT has been abundantly detected in khat samples across the country (Daba et al. 2011; Mekonen et al. 2017; Regassa et al. 2020). The levels of this congener were substantially higher than the maximum residue limit (MRL) set by the European Commission (EC) (Daba et al. 2011). Unlike other leafy food crops, the water content of khat is relatively low and it is consumed raw and unwashed. Most chewers consume khat daily and excessively. According to Mekonen et al. (2017), the mean daily khat consumption among khat chewers in southwestern Ethiopia is 19.6 g per kg body weight.

While most studies on khat focus instead on psychotic, pharmacological, drug and medical aspects as well as research on the political, socioeconomic and development effects in the horn of Africa and Arabian Peninsula (Carrier and Klantschning 2016; Cafer and Rikoon 2017; Badedi et al. 2020), health risks associated with illicit OCP use in khat cultivation systems have been scarcely studied (Ligani and Hussen 2014; Mekonen et al. 2017; Regassa et al. 2020). Thus, assessing concentrations of DDT and HCH congeners in khat originated from different khat-based agroecosystems, and assessing the health risks associated with the chewing practice of DDT and HCH contaminated khat in South Wollo, are of paramount importance. Therefore, the main aim of the present study was, for the first time, to quantify the levels of DDT and HCH congeners in five popular khat varieties (Gerba, Hayq-Gallissa, Kemissie-Bekela, Bahir Dar, and Kemissie-Normal). Other aims were (i) to estimate dietary intake of DDTs and HCHs and (ii) to assess cancer and non-cancer risks associated with DDT and HCH congeners through the consumption of khat.

## Materials and methods

### Study area

Dessie, a town in north-central Ethiopia, is located 400 km away from the capital Addis Ababa, Ethiopia. Based on the population projection of Ethiopia, Dessie had a total population of 212,436 in 2014 (CSA 2012). It is located at an altitude of 2,470 m above sea level. The town is divided into five sub-cities for administrative purposes. Street markets are the most common and preferred places to sell khat. Saleswomen and men of street markets look for strategic places, usually along roadsides.

### Khat sample collection and processing

A total of 25 marketing areas across the town were selected based on the varieties of khat they retail (locally known as Gerba, Hayq-Gallissa, Bahir Dar, Kemissie-Bekela and Kemissie-Normal). These five local khat varieties commonly originate from four prominent khat producing areas (Bati, Hayq, Bahir Dar and Kemissie, respectively) in Amhara regional state, Ethiopia. For each variety, 21 khat subsamples were procured (105 in total). Among one variety, each three subsamples were combined, resulting in seven composite khat samples per variety (35 in total). These were then packed in perforated paper envelopes and left to dry under shade for seven days. The moisture contents varied between 49 and 51.7%. After that samples were transported to Justus Liebig University Giessen, Germany, for DDT and HCH analysis.

### Chemicals and reagents

Standards (purity) of *o*,*p*′*-*DDD (97.5%), *o*,*p*′*-*DDE (99%), *o*,*p*′*-*DDT (99.5%), *p*,*p*′*-*DDD (99.5%), *p*,*p*′*-*DDE (98%), *p*,*p*′*-*DDT (99.5%), ^13^C-o,*p*′-DDT (100%) and γ-HCH (98.6%) were purchased from Dr. Ehrenstorfer GmbH (Augsburg, Germany). α-HCH (≥ 98%) and δ-HCH (≥ 98%) were purchased from Sigma-Aldrich (St. Louis, MO, USA). β-HCH (99.5%) was obtained from Institute of Industrial Organic Chemistry (Warsaw, Poland). *p*,*p*′-DDD-D8 (99.7%), *p*,*p*′-DDE-D8 (99.4%) and α-HCH-D6 (99.2%) were purchased from CDN Isotopes (Pointe Claire, Canada). ^13^C-*p*,*p*′-DDT (99%) was purchased from Cambridge Isotope Laboratories Inc. (Andover, MA, USA). Purity was considered when producing stock solutions of standards. Extraction solvents, methanol (100%), and acetonitrile (99.9%), both HPLC grade, were obtained from VWR International (Radnor, PA, USA), as well as the extraction salt MgSO_4_. Sodium citrate dibasic sesquihydrate (≥ 99%) was purchased from Sigma-Aldrich. Tri-sodium citrate dihydrate (≥ 99.5%) was purchased from Th. Geyer GmbH & Co. KG (Renningen, Germany). The primary-secondary amine (PSA) was obtained from Supelco (Bellefonte, PA, USA). Salts for SPME solution, sodium chloride (≥ 99.5%) and calcium chloride were purchased from Carl Roth GmbH & Co. KG (Karlsruhe, Germany) and Merck KGaA (Darmstadt, Germany), respectively. All salts were of analytical grade or better. Ultra pure water was produced using Milli-Q A10 water purification system (Merck KGaA).

### QuEChERS extraction

Extraction was carried out using a QuEChERS protocol based on the development of Anastassiades et al. (2003): 1 g of homogenized khat sample was placed into a 50 mL screw‐cap glass centrifuge tube together with 10 mL of ultrapure water and vortexed for 10 s. After waiting 10 min for the sample to rehydrate, 15 mL acetonitrile was added and samples were shaken on a horizontal shaker (Swip KS-10, Edmund Bühler GmbH, Bodelshausen, Germany) for 15 min at 200 rpm. Afterwards, 4 g MgSO_4_, 1 g NaCl, 1 g sodium citrate (tribasic) dihydrate and 0.5 g sodium citrate (dibasic) sesquihydrate were added and vortexed again for 10 s, before it was centrifuged for 10 min at 1,000 rpm (207.2 *g*) (Rotanda 460 R, Hettich AG, Bäch, Switzerland). After centrifugation, 8 mL of organic supernatant was transferred into a centrifuge tube containing 1.2 g MgSO_4_ and 0.2 g PSA and was vortexed for 10 s. The tube was then centrifuged at 2,500 rpm (1,295 *g*) for 10 min. A 4 mL aliquot of the supernatant was transferred into a 20 mL brown glass head space vial, 2 µL of internal standard mix (see Table S1) were added and vortexed. The sample was evaporated to dryness under a gentle stream of nitrogen and immediately resolubilized with 100 µL of methanol after which it was vortexed for 5 s, and 10 mL of salt solution (1.47 g CaCl_2_ and 200 g NaCl in 1 L ultrapure water) were added. The vial was closed firmly with a septum screw cap. The extraction was done in triplicate per sample and separately for each group of analytes (DDX and HCH) resulting in six extractions per sample.

### SPME extraction and GC–MS analysis

Pesticide analysis in khat was carried out with a Trace 1310 gas chromatograph (Thermo Fisher Scientific, San Jose, CA, USA) equipped with a PTV‐injector operated in constant temperature mode (260 °C), a CombiPAL autosampler (CTC Analytics AG, Zwingen, Switzerland) equipped with a SPME fibre assembly, and an ISQ 7000 mass spectrometer (Thermo Fisher Scientific). For HCH and DDX analysis different SPME fibers were used: PDMS/DVB 65 µm and PDMS 100 µm, respectively (Sigma-Aldrich). SPME extraction of prepared samples started with a heat up phase of 5 min to 80 °C in the agitator, followed by head space extraction at the same temperature for 30 min and 60 min for DDT and HCH, respectively. After extraction, the fibre was thermally desorbed in splitless mode in the GC injector for 3 min, after which it switched back to a split flow of 30 mL min^−1^. At the start and end of each SPME sample cycle, the fibre was desorbed in a needle heater for 7.5 min at 270 °C. Chromatographic separation was conducted on a fused silica capillary column (TG-XLBMS: 60 m, 0.25 mm inner diameter; 0.25 μm coating thickness; Thermo Fisher Scientific). Helium (≥ 99.999%) was used as carrier gas at a constant flow of 1.0 mL min^−1^. The initial oven temperature was set at 90 °C and held for 3 min. The temperature was ramped to 150 °C at a rate of 15 °C min^−1^. Then, it was ramped to 280 °C at a rate of 5 °C min^−1^ and held for 3 min. Full scan analysis was used to determine the chromatographic and MS characteristics of the analytes. Quantification was performed in selected ion monitoring (SIM) mode based on one target and one qualifier ion. For a complete list of ions and retention times used, refer to Table S2. The peak areas of DDT and HCH congeners in khat samples were corrected with their respective internal standards and the according concentration was determined by interpolation of the relative peak areas for each pesticide to standard peak areas of the calibration curve.

### Method validation

Linearity, precision and recovery were evaluated using a seven-level matrix matched calibration curve at concentrations of 0.01, 0.05, 0.1, 0.5, 1.0, 1.5 and 2.0 µg kg^−1^ for DDX, and 0.1, 0.5, 1.0, 1.5, 2.0, 2.5 and 3.0 µg kg^−1^ for HCH. For each level, dried and ground leaves of cherry laurel (*Prunus laurocerasus*) as surrogate for uncontaminated khat leaves were extracted and re-dissolved in salt solution and methanol, like ordinary samples. Prior to measurement, calibration and internal standards were added. Recovery and precision of the method were determined by extraction and analysis of a (cherry laurel) matrix sample spiked with each analyte at 100 µg kg^−1^ in five replicates. For determination of limits of detection (LOD) and limits of quantitation (LOQ), five blank samples containing 10 mL salt solution and 100 µL methanol were analysed. The integrated noise at the retention times of the analytes was multiplied by 3 and 10 resulting in LODs and LOQs, respectively.

### Health risk assessment

For the evaluation of carcinogenic effects of consuming DDT and HCH contaminated khat, cancer risk (CR) was employed according to US EPA (2000, 2001), while non-carcinogenic effects were assessed by comparing daily exposure with the reference dose set by the United States Environmental Protection Authority (US EPA 2000, 2001). The 50th (median or P_50_) and 95th (P_95_) percentile concentrations instead of the mean values were used to estimate daily intakes and compute hazard index (HI) and CR, whereas the P_95_ is supposed to represent the worst-case scenario or high-end exposure probability.

#### Dietary intake estimation (exposure)

The exposure depends on both the DDT and HCH concentrations of khat and the daily consumption pattern. According to Duresso et al. (2016), a typical khat chewing session is characterized by the consumption of 100 to 500 g fresh khat leaves. Since no information regarding per capita consumption of khat among khat chewers in South Wollo and beyond is available, 100 g (low-end consumption probability) and 500 g (high-end consumption probability) were considered as daily portions. To estimate exposure, the dry weight (under shade) was converted into wet weight [see Sect. 2.2]. The average weight of an adult was set to 60 kg. The dietary intake (exposure) was estimated with the following formula:

$${\text{EDI}} = Q_{{\text{F}}} \times C_{{\text{P}}} /{{\text{BW}}}$$

where EDI is estimated daily intake [ng (kg BW)^−1^ day^−1^]; *Q*_F_ represents daily intake of khat [g day^−1^]; *C*_P_ represents median levels of individual OCPs in khat [ng g^−1^] (fresh weight) and BW represents average body weight [kg].

#### Hazard index (HI)

The non-cancer health risk associated with individual intakes of *p*,*p*′-DDT, β*-*HCH, δ*-*HCH and γ*-*HCH through the consumption of khat was based on HI. The HI is the ratio of estimated daily intake to a reference dose level. If the ratio is less than 1, the exposed population is unlikely to experience obvious adverse effects. The HI was calculated as follows (US EPA 2000, 2001):

$${\text{HI }} = {\text{ EDI}}/{\text{RfD}}\;{\text{or}}\;{\text{EDI}}/{\text{ADI}}$$

where ADI is acceptable (tolerable) daily intake [ng (kg BW)^−1^ day^−1^] and RfD is the oral reference dose [ng (kg BW)^−1^ day^−1^]. The RfD for *p*,*p*′-DDT was set to 500 ng (kg BW)^−1^ day^−1^ as suggested by US EPA (1987) and the ADI for β*-*HCH, δ*-*HCH, and γ*-*HCH was set to 300 ng (kg BW)^−1^ day^−1^ as proposed by US EPA (2017).

#### Cancer risk

To calculate CR, a formula described by US EPA (2000, 2001) was used. A risk level greater than 10^–4^ is considered unacceptable, while the area of concern is set between 10^–4^ and 10^–6^.

$${\text{CR}} = {C_{n}} \times I \times 10^{ - 3} \times {\text{EFr}} \times {\text{ED}} \times {\text{CSF}}_{{\text{o}}} /\left( {{\text{BW}} \times {\text{AT}}} \right)$$

where *C*_n_ represents the median levels of *p*,*p*′-DDT, total DDT and HCH isomers in khat [mg kg^−1^]; *I* is chewing rate [g person^−1^ day^−1^]; EFr is exposure frequency (365 days year^−1^); ED is exposure duration (47 years, average life expectancy minus 18 years); BW is the average body weight (adult, 60 kg); AT is the average exposure time for non-carcinogens (365 days year^−1^ × number of exposure years). CSF_o_ is the cancer oral slope factor for individual OCPs, and was set to 0.34 for *p*,*p*′-DDT, 1.8 for β*-*HCH and δ*-*HCH, and 1.3 for γ*-*HCH (mg kg^−1^ day^−1^)^−1^ as proposed by US EPA (2017).

### Statistical analysis

Statistical analysis was carried out with SPSS database version 23 (IBM Inc., NY, USA). Whenever concentrations were below limit of detection (< LOD) or below limit of quantitation (< LOQ), LOD/2 and LOQ/2 were used as arbitrary concentrations to compute mean, P_50_ and P_95_, only when the frequency of occurrence was 60% or more (WHO 2008). The normal distribution and homogeneity of variance were tested using Kolmogorov–Smirnov’s and Levene’s tests, respectively. Due to non-normal distributions of data, Kruskal–Wallis test was used in combination with Dunn’s multiple comparison test (non-parametric post-hoc) to evaluate statistical difference in median contents of OCPs among khat varieties. Statistical significance was established at *p* < 0.05.

## Results and discussion

### Method validation and concentrations of OCPs

The method validation results are indicated in Table S3. LODs and LOQs for DDT congeners were 0.1 and 0.3 µg kg^−1^, respectively. For HCH, LODs were 0.15–0.45 µg kg^−1^, and LOQs were 0.5–1.5 µg kg^−1^. Recovery rates were satisfactory, ranging from 86.72 to 114.75% for DDX, and from 97.33 to 111.12% for HCH. Concentrations of *o*,*p*′*-*DDD, *o*,*p*′*-*DDE, *o*,*p*′*-*DDT, *p*,*p*′*-*DDD, *p*,*p*′*-*DDE, *p*,*p*′*-*DDT and total DDT ranged from < 0.1 to 5.24, < 0.1 to 4.13, < 0.1 to 64.2, 0.6 to 154, 1.47 to 14.9, 3.34 to 1,193 and 6.2 to 1,433 µg kg^−1^, respectively (Table 1). The frequencies of occurrence were 82.9%, 85.7% and 94.3% for *o*,*p*-DDD, *o*,*p*′-DDE and *o*,*p*′-DDT, respectively, and 100% each for *p*,*p*′-DDD, *p*,*p*′-DDE and *p*,*p*′-DDT. Regardless of the varieties, samples were dominated by *p*,*p*′-DDT. Other congeners’ concentrations, such as *o*,*p*′-DDD and *o*,*p*′-DDE, were relatively low (Table 1). The occurrence of extremely high and low *p*,*p*′-DDT concentrations in Hayq-Gallissa khat might be due to different pesticide management practices among Hayq-Gallissa growing farmers (own observations). Furthermore, the present study revealed that 100% of the khat samples contained *p*,*p*′*-*DDT, but only 25.7% of these samples exhibited total DDT levels above the MRL set by the EC (2005). Conversely, earlier studies in eastern and southwestern Ethiopia revealed lower frequencies of *p*,*p*′-DDT occurrence in khat samples, ranging from 61.7 to 66.7% (Daba et al. 2011; Mekonen et al. 2017). The median concentrations of *p*,*p*′-DDT in khat follows this order: Kemissie-Normal (69.5 µg kg^−1^) > Kemissie-Bekela (25.4 µg kg^−1^) > Hayq-Gallissa (22.7 µg kg^−1^) > Gerba (15.5 µg kg^−1^) > Bahir Dar (3.75 µg kg^−1^) (Table 1). The median concentrations of *p*,*p*′-DDT and total DDT in Kemissie-Normal khat were significantly higher than in Bahir Dar. Bahir Dar khat displayed a relatively low median level for *p*,*p*′-DDT. This is ascribed to the preference for Endosulfan among khat and vegetable farmers in Bahir Dar (Sishu et al. 2020). A similar trend was observed for total DDT.

**Table 1:** Mean ± standard error (SE), P_50_ (P_95_), range and frequency of occurrence (%) of organochlorine pesticides in khat samples (*n* = 7) collected from markets in South Wollo, Ethiopia, 2019

OCP		Gerba	Hayq-Gallissa	Kemissie-Bekela	Bahir Dar	Kemissie-Normal
*o*,*p*′-DDD						
	Mean ± SE	1.19 ± 0.36	1.59 ± 0.72	1.22 ± 0.32	0.70 ± 0.32	1.08 ± 0.34
	P_50_ (P_95_)	0.74 (2.68)	0.89 (4.60)	1.09 (2.44)	0.49 (2.02)	1.31 (2.14)
	Range	0.31–2.93	< 0.1–5.24	< 0.1–2.78	< 0.1–2.49	< 0.1–2.33
	Frequency	100	85.7	85.7	71.4	71.4
*o*,*p*′-DDE						
	Mean ± SE	0.87 ± 0.44	0.8 ± 0.2	1.04 ± 0.30	0.88 ± 0.55	0.37 ± 0.10
	P_50_ (P_95_)	0.46 (2.69)	0.59 (1.57)	0.71 (2.29)	0.42 (3.1)	0.35 (0.75)
	Range	< 0.1–3.46	< 0.3–1.64	0.45–2.54	< 0.1–4.13	< 0.1–0.8
	Frequency	85.7	85.7	100	71.4	71.4
*o*,*p*′-DDT						
	Mean ± SE	3.08 ± 0.45	15.1 ± 8.5	5.34 ± 0.91	0.95 ± 0.29	10.2 ± 2.06
	P_50_ (P_95_)	3.3^ab^ (4.64)	3.79^ab^ (50.0)	6.36^ab^ (7.89)	0.85^b^ (1.92)	11.3^a^ (16.0)
	Range	1.45–4.96	1.54–64.2	1.45–8.08	< 0.1–1.96	2.88–16.35
	Frequency	100	100	100	71.4	100
*p*,*p*′-DDD						
	Mean ± SE	2.51 ± 0.43	27.3 ± 21.2	4.48 ± 0.52	1.41 ± 0.43	9.17 ± 1.96
	P_50_ (P_95_)	2.09 (4.32)	6.24 (112)	4.48 (5.87)	0.99 (3.14)	11.0 (14.7)
	Range	1.49–4.89	2.06–154.4	1.98–5.96	0.60–3.85	2.80–15.03
	Frequency	100	100	100	100	100
*p*,*p*′-DDE						
	Mean ± SE	2.60 ± 0.37	4.55 ± 1.76	3.71 ± 0.46	3.09 ± 0.45	4.42 ± 0.61
	P_50_ (P_95_)	2.49 (4.02)	2.65 (11.8)	3.06 (5.44)	2.63 (4.92)	3.84 (6.59)
	Range	1.47–4.46	1.82–14.89	2.48–5.74	1.87–5.44	2.67–6.82
	Frequency	100	100	100	100	100
*p*,*p*′-DDT						
	Mean ± SE	13.4 ± 2.05	199 ± 166	23.5 ± 3.82	4.21 ± 0.34	66.2 ± 16.4
	P_50_ (P_95_)	15.5^ab^ (18.3)	22.7^ab^ (864)	25.4^a^ (34.4)	3.75^b^ (5.27)	69.5^a^ (118)
	Range	3.81–18.76	4.38–1193	10.51–34.61	3.24–5.35	15.98–123.5
	Frequency	100	100	100	100	100
Total DDT						
	Mean ± SE	21.7 ± 3.28	247 ± 198	37.6 ± 5.05	9.42 ± 1.55	90.6 ± 21.1
	P_50_ (P_95_)	23.8^ab^ (31.1)	33.2^ab^ (1040)	42.8^ab^ (51.6)	7.78^b^ (15.7)	96.2^a^ (157)
	Range	8.61–33.65	10.5–1433	16.53–51.61	6.2–17.96	26.15–162.8
α*-*HCH						
	Mean ± SE	1.18 ± 0.11	1.07 ± 0.22	2.52 ± 0.46	1.59 ± 0.22	1.58 ± 0.30
	P_50_ (P_95_)	0.5 (1.62)	1.1 (2.47)	2.01 (4.0)	1.46 (2.46)	1.16 (2.73)
	Range	< 1–1.68	< 1–2.66	1.16–4.16	1.18–2.85	< 1–2.77
	Frequency	42.9	57.1	100	100	57.1
β*-*HCH						
	Mean ± SE	47 ± 6.4	36.4 ± 6.51	33.2 ± 3.92	37.6 ± 2.06	41.3 ± 4.19
	P_50_ (P_95_)	41.5 (72.0)	44.7 (54.0)	27.7 (48.5)	34.9 (46.0)	38.3 (57.5)
	Range	27.58–80.39	32.29–54.17	23.0–49.58	33.26–47.21	30.14–62.08
	Frequency	100	100	100	100	100
δ*-*HCH						
	Mean ± SE	15.2 ± 0.72	16.3 ± 0.99	14.6 ± 1.1	22.8 ± 1.56	17.1 ± 0.88
	P_50_ (P_95_)	15.9 (16.6)	16.1 (20.2)	14.1 (17.9)	23.0 (27.8)	16.2 (20.7)
	Range	11.2–16.67	14.6–21.32	10.5–18.23	18.09–28.42	15.04–21.17
	Frequency	100	100	100	100	100
γ*-*HCH						
	Mean ± SE	2.98 ± 0.3	4.5 ± 1.53	4.15 ± 1.01	3.20 ± 0.13	2.90 ± 0.16
	P_50_ (P_95_)	2.93 (4.04)	2.94 (3.52)	3.66 (8.26)	3.23 (3.62)	3.0 (3.26)
	Range	1.92–4.07	2.29–3.57	1.77–9.90	2.63–3.72	2.01–3.36
	Frequency	100	100	100	100	100
Total HCH						
	Mean ± SE	60.7 ± 6.43	52.5 ± 8.78	48 ± 3.18	63.1 ± 3.54	61.4 ± 4.02
	P_50_ (P_95_)	53.9 (85.9)	62.3 (73.2)	47.1 (59.1)	60.5 (77.7)	57.5 (75.3)
	Range	41.54–94.15	50.7–73.41	40.17–61.67	54.75–78.83	48.20–80.49
*o*,*p*′-DDT/*p*,*p*′-DDT						
	Mean ± SE	0.25 ± 0.04	0.21 ± 0.04	0.23 ± 0.03	0.24 ± 0.07	0.18 ± 0.03
	Range	0.16–0.47	0.05–0.35	0.14–0.38	0.01–0.53	0.10 ± 0.27

Values with different letters within a row are significantly different at *p* < 0.05 level

Mean, P_50_ and P_95_ values are given in µg kg^−1^ dry matter

Compared with data of similar studies across Ethiopia, concentrations of total DDT in khat in South Wollo were comparable to those in Sidama (16.7–44.8 µg kg^−1^), and lower than to those in Jimma (41.4–149 µg kg^−1^), Gelemso (755 µg kg^−1^) and Haramaya (111 µg kg^−1^) (Daba et al. 2011; Ligani and Hussen 2014; Mekonen et al. 2017; Regassa et al. 2020). However, comparison of concentration data is qualitative at best, because available data formats (mean vs. median and discrepancies in number of samples) were quite variable among the studies.

**Fig. 1:**
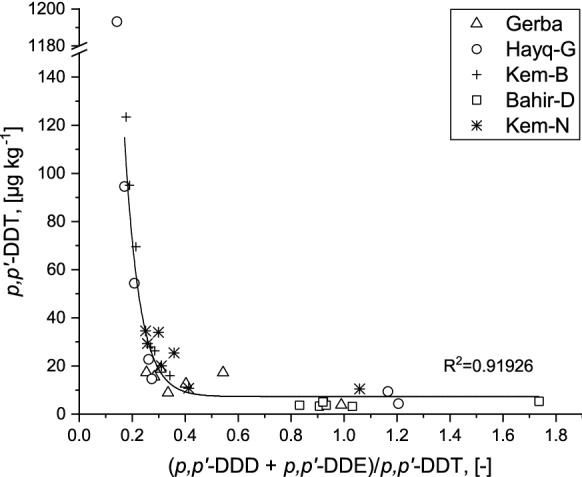
Scatter plot of *p*,*p*′-DDT concentration [µg kg^−1^] and ratio between transformation products of and *p*,*p*′-DDT itself for five different khat varieties including an exponential fit indicating the dependence of the TPs from the parent compound

The ratios between the parent OCPs and their transformed products have been generally used to discern whether the pollution is from past usage or recent application. For DDT, (*p*,*p*′-DDD + *p*,*p*′-DDE)/*p*,*p*′-DDT is a popular diagnostic in that matter (Calamari et al. 1991; Xiaofei et al. 2008). A value greater than one is indicative of past DDT application, while a value less than one could be associated with recent usage (Calamari et al. 1991; Bosch et al. 2015). In the present study, these values were found to be lower than one for 85% of khat samples (Fig. 1), indicating ubiquitous recent contamination with little contribution due to historical usage. Regarding the ratios of (*p*,*p*′-DDD + *p*,*p*′-DDE)/*p*,*p*′-DDT, estimated from median concentrations, Kemissie-Normal showed a very low value (0.21), followed by Gerba (0.29). Yet, the other varieties have also exhibited very low ratios except Bahir Dar (0.97). In comparison, relatively higher diagnostic ratios in khat were obtained across various locations in Ethiopia (0.73–1.27; Ligani and Hussen 2014; 4.1–7.0; Mekonen et al. 2017). Based on such comparisons, there might be intensive recent use of DDT in khat cultivation and vector control in South Wollo. The strong exponential correlation in Fig. 1 (*R*^2^ approx. 0.92), suggests that the transformation products’ concentrations and that of the parent compound are very much dependent of each other. This indicates that the occurring transformation products were autochthonously formed from applied DDT rather than stemming from external sources such as long-range atmospheric transport. Yet cannabis, an illicit drug crop with higher stimulant property, exhibited lower levels of OCPs from legal market in USA (Russo 2016). In fact, the chemical signatures obtained in cannabis may reflect historical OCP usage. According to a study by Tsakiris et al. (2015), a low ratio of *p*,*p*′-DDE to *p*,*p*′-DDT in milk might be partly explained by extensive use of dicofol as an acaricide: DDT is used as a raw material in the production of dicofol, and DDT may occur as a dicofol impurity ranged from 0.1 to 10% (Rasenberg and van de Plassch 2002; Zhong et al. 2003). However, dicofol type DDT pollution is characterized by a high ratio of *o*,*p′*-DDT to *p*,*p′*-DDT (Qiu et al. 2005; Yang et al. 2008; Qiu and Zhu 2010). In the present study, *o*,*p*′-DDT to *p*,*p*′-DDT ratios were comparable with that of the technical DDT mixture (Table 1), which amounts to approx. 20:80 (Metcalf 1955). Thus, khat samples are probably negligibly impacted by the usage of dicofol (Yang et al. 2008; Yohannes et al. 2014).

The isomers of HCH were also detected in different concentrations among the khat varieties under investigation (Table 1). Their concentrations in khat samples ranged from < 1 to 4.16 µg kg^−1^ for α-HCH, 23 to 80.4 µg kg^−1^ for β*-*HCH, 10.5 to 28.4 µg kg^−1^ for δ*-*HCH and 1.92 to 9.9 µg kg^−1^ for γ*-*HCH. α-HCH was hardly quantified in approximately 30% of the khat samples, in the rest of the samples, it was present in very small quantities (1.16–4.16 µg kg^−1^; Table 1). The highest median concentrations of β*-*HCH (44.7 µg kg^−1^) and total HCH (62.3 µg kg^−1^) were obtained from Hayq-Gallissa variety, whereas the lowest concentrations were detected in Kemissie-Bekela (27.7 and 47.1 µg kg^−1^, respectively). With the exception of Kemissie-Normal, the median concentration of total HCH was higher than that of total DDT in all other khat varieties. This might be explained by superior accumulation of HCHs on plant surfaces due to higher vapor pressure than those of DDTs (Walker et al. 1999). Another reason could be a difference in the amount of application over the past decades. For example, Chourasiya et al. (2015) have noted that the high HCH concentration in vegetables could be ascribed to the excessive production and usage of technical HCH (ten times more than that of technical DDT) before its ban in 1983.

**Fig. 2:**
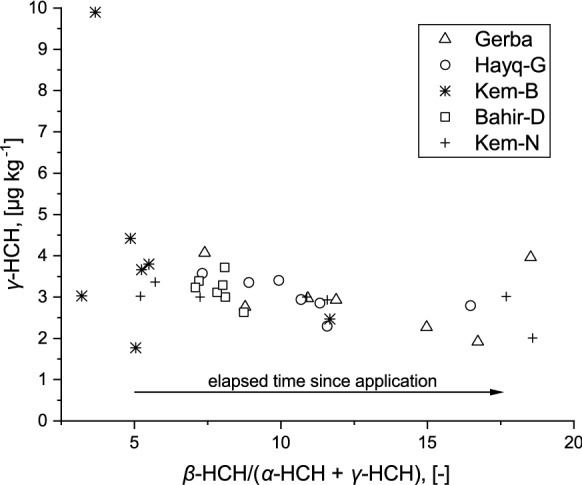
Scatter plot of γ-HCH concentration [µg kg^−1^] and ratio of the most persistent isomer (β-HCH) to the other less persistent isomers (α- and γ-HCH). Rising ratios indicate the elapsing time since application. With increasing time, β-HCH becomes the most prominent isomer, whereas the γ-HCH concentration slowly diminishes

Among the four isomers of HCH, β*-*HCH was dominant in khat samples, but α-HCH hardly occurred. Due to the fact that khat is only cultivated in East Africa and the Arabian Peninsula, conventional crops were chosen for comparison. Compared with the levels of β*-*HCH in conventional food crops in Pakistan (0.37–1.96 µg kg^−1^; Aamir et al. 2018), and India (0.38–2.58 µg kg^−1^; Pathak et al. 2016), the β*-*HCH values obtained in the present study were high. On the other hand, the median concentrations of total HCH in khat samples were approximately two or more orders of magnitude lower than in selected conventional crops in Spain (4,670–32,270 µg kg^−1^; Barriada-Pereira et al. 2005), and India (322–1,235 µg kg^−1^; Srivastava et al. 2011). Technical HCH and lindane were the two popular formulations of HCH. While the latter is mainly γ*-*HCH (99%), the former product shows congener ratios of approximately 11.8 and 4.6–5.8 for α/β-HCH and α/γ-HCH, respectively (Zhang et al. 2011; Niu et al. 2013). It is important to note that the ratio changes with time as the isomers degrade and dissipate at different rates (Middeldorp et al. 1996). In comparison to the other isomers, β*-*HCH is more stable and resistant to microbial degradation (Willett et al. 1998). In the present study, β*-*HCH was very dominant in khat samples; the proportion of β*-*HCH to total HCH ranged from 57.7 to 77%, implying the transformation of the parent isomers to β*-*HCH over the years. Predominance of β*-*HCH was in accordance with the vegetable results reported by Kumar and Mukherjee (2012), Fang et al. (2015) and Chourasiya et al. (2015). The ratio of β-/(α + γ)-HCH can also be used to identify the possible input sources of HCHs, where a ratio of greater than 0.5 indicates past input (Yi et al. 2013). In the present study, this ratio varied from 3.2 to 18.5. In addition, extremely high β-HCH concentrations as compared to γ-HCH were found in khat samples (Table 1; Fig. 2), and it suggested the input of past technical HCH (Li et al. 2006).

### Health risk assessment

Estimation of the level of DDT and HCH exposure to different life stages and gender are of great importance for observing the potential health risks. Because khat is a stimulant, consumption by children should be negligible. And since it’s rarely consumed by women, in the present study, DDT and HCH exposure was assessed for adult men only. As shown in Table 2, EDI values for different khat varieties were presented as median. The highest intakes of *p*,*p*′-DDT (290 ng (kg BW)^−1^ day^−1^) and total DDT (401 ng (kg BW)^−1^ day^−1^) were obtained from high-end consumption probability of Kemissie-Normal khat. These EDI values were far lower than a value obtained in Jimma in southwestern Ethiopia (2000 ng (kg BW)^−1^ day^−1^; Mekonen et al. 2017). On the other hand, comparable EDI values would be obtained from the consumption of khat collected from various locations across Sidama in southern Ethiopia (24.2–325 ng (kg BW)^−1^ day^−1^; Ligani and Hussen 2014). Again, it should be noted that this is a mere qualitative comparison, since consumption rate and data format were variable among these three studies. For example, Mekonen et al. (2017) investigated exposure to DDT from khat consumption by taking 19.2 g (kg BW)^−1^ as a daily consumption rate. On the contrary, the EDIs of *p*,*p*′-DDT and total DDT from the consumption of Kemissie-Normal khat are not only higher than those obtained from market-based data in India (vegetarian diet (2001)): 36.7 ng (kg BW)^−1^ day^−1^ (Battu et al. 2005), Sweden: 8.72 ng (kg BW)^−1^ day^−1^ (Darnerud et al. 2006), and USA: 4.38–8.72 ng (kg BW)^−1^ day^−1^ (Schecter et al. 2010). The EDIs are also higher than those that resulted from consumption of staple cereals and cereal-based foods in Poland (30 ng (kg BW)^−1^ day^−1^; Roszko et al. 2020), Russia (31 ng (kg BW^−1^ day^−1^; Polder et al. 2010) and Pakistan (40 ng (kg BW)^−1^ day^−1^; Mehmood et al. 2017). Similarly, dietary intake of total DDT with vegetables were found to be relatively low, ranging from 1.13 to 8.87 ng (kg BW)^−1^ day^−1^ (Fang et al. 2015; Bolor et al. 2018).

**Table 2:** Estimated dietary intakes [ng (kg BW)^−1^ day^−1^] of OCPs and hazard index (HI) of potential non-carcinogenic effects for *p*,*p*-DDT, β-HCH, δ-HCH and γ*-*HCH from the consumption of 100 g (low-end consumption probability) and 500 g (high-end consumption probability) fresh khat leaves

OCP	Variety										RfD or ADI [ng (kg BW)^−1^ day^−1^]
	Gerba		Hayq-Gallissa		Kemissie-Bekela		Bahir Dar		Kemissie-Normal		
	100 g	500 g	100 g	500 g	100 g	500 g	100 g	500 g	100 g	500 g	
*o*,*p*′-DDT	2.75	13.7	3.16	15.8	5.30	26.5	0.7	3.52	9.39	47.0	–
*p*,*p*′-DDD	1.74	8.70	5.2	26.0	3.73	18.7	0.83	4.13	9.13	45.7	–
*p*,*p*′-DDE	2.08	10.4	2.21	11.0	2.55	12.7	2.19	11.0	3.2	16.0	–
*p*,*p*′-DDT	12.9	64.8	19.0	94.8	21.1	105.7	3.12	15.6	57.9	289.6	300^a^
Total DDT	19.8	99.1	27.7	138.3	35.7	178.3	6.49	32.4	80.2	400.9	–
β-HCH	34.6	173.0	37.2	186.1	23.0	115.2	29.0	145.2	31.9	159.6	300^b^
δ-HCH	13.2	66.1	13.4	67.0	11.7	58.7	19.1	95.7	13.5	67.4	300^b^
γ-HCH	2.44	12.2	2.45	12.3	3.05	15.3	2.70	13.5	2.50	12.5	300^b^
Total HCH	45.0	224.8	51.9	259.6	39.2	196.1	50.4	252.2	47.9	239.6	–
HI (*p*,*p*′-DDT)	0.026	0.130	0.038	0.191	0.043	0.213	0.006	0.030	0.116	0.578	
HI (β-HCH)	0.115	0.574	0.123	0.617	0.077	0.383	0.097	0.483	0.106	0.530	
HI (δ-HCH)	0.044	0.221	0.047	0.224	0.039	0.196	0.063	0.317	0.045	0.226	
HI (γ-HCH)	0.008	0.041	0.008	0.041	0.010	0.052	0.010	0.051	0.009	0.045	

Estimation based on 50th percentile concentration. The dietary intakes for α-HCH were not estimated due to very low concentrations

^a^US EPA (1987)

^b^US EPA (2017)

The daily intakes of β*-*HCH through khat consumption by adult men were estimated to vary from 23 to 186 ng (kg BW)^−1^ day^−1^ (Table 2). According to Shen et al. (2013), the per capita vegetables consumption by adult females in south China was estimated as 279 g day^−1^ and the daily intake of β-HCH associated with the consumption of this food group was two- to three-fold lower than those obtained in the present study. These results are worrisome, as the possibility exists that khat consumers might be additionally exposed to high DDT and HCH concentrations from potentially contaminated food items. Table 2 also shows the non-carcinogenic risks associated with total DDT, β-HCH, δ-HCH and γ-HCH intakes through the consumption of khat. For α-HCH, the calculation of HI was not possible due to its infrequent detection in the samples. The HI values not only depend on OCP concentration but also on average body weight and quantity of food or stimulant consumed: both ultimately affect an individual's level of risk from OCPs (Shen et al. 2013). Individuals with low body weight are more vulnerable to toxicological safety limits, which becomes obvious if exposure data of adults and children is compared (Ferreira et al. 2020). For DDT and HCH, no international organization including the WHO has set acute reference doses which leads to difficulties in estimating an acute or short-term health risk assessment. Overall, the potential non-carcinogenic risk of β*-*HCH (HI: 0.077–0.530) was higher than that of *p*,*p*′-DDT (HI: 0.006–0.578) at P_50_ concentration. However, both were below the threshold (HI = 1) stipulated by the US EPA (2000, 2006). All previous and recent DDT related studies in khat would have displayed lower HI values from a low consumption rate of 100 g day^−1^ (Ligani and Hussen 2014; Mekonen et al. 2017; Regassa et al. 2020). Since khat farmers use DDT even when there are no diseases or pests present, the HI could be further reduced by avoiding such misuse. To the best of our knowledge, this is the first study about the status of HCH in khat and associated potential health risk from khat chewing practice. Thus, comparison with similar studies was impossible. However, comparably low HI values were obtained for β*-*HCH and γ*-*HCH from the consumption of various conventional vegetables across the developing countries (0.14, Chourasiya et al. 2015; 0.052, Akoto et al. 2015; 0.004–0.41, Fang et al. 2015; 0.00271–0.000473, Bempah et al. 2016; 0.28–0.37, Buah-Kwofie et al. 2019).

**Fig. 3:**
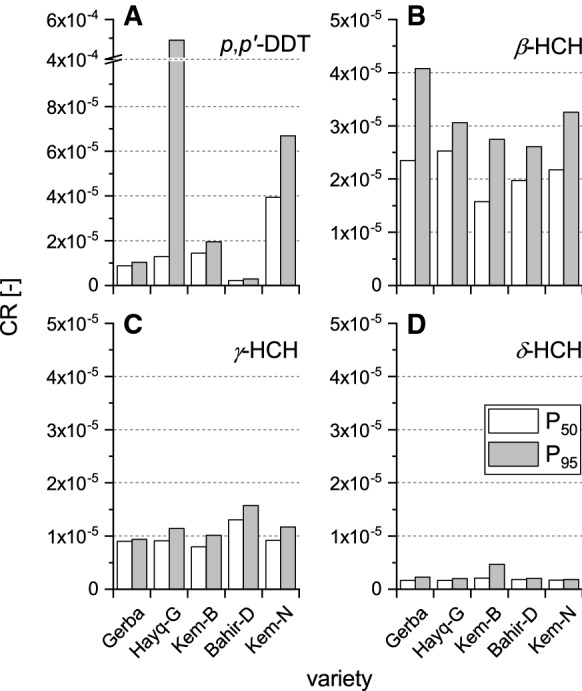
Cancer risk values for *p,p'-*DDT, β-HCH, δ-HCH and γ-HCH through the consumption of 100 g fresh khat leaves (low-end consumption probability) for five khat varieties at P_50_ and P_95_ concentrations

The cancer risks associated with *p*,*p*′-DDT, β*-*HCH, δ*-*HCH and γ*-*HCH intakes through the consumption of 100 g fresh khat leaves (low-end consumption probability), at P_50_ and P_95_, for adult in South Wollo are presented in Fig. 3. All khat varieties showed a CR value greater than 10^–6^. For example, Kemissie-Normal displayed a CR of 4.53 × 10^–5^ (P_50_) and a CR of 7.69 × 10^–5^ (P_95_) for *p*,*p*′-DDT (Fig. 3a). If we assume a chewing rate of 100 g day^−1^ with the present *p*,*p*′-DDT concentration in Kemissie-Normal khat, the cancer risk at P_50_ concentration results in almost four chances per 100,000. At P_95_ concentration, this equates to seven per 100,000. Similarly, cancer risk was also associated with the consumption of Hayq-Gallissa variety, which shows a CR of 1.29 × 10^–5^ and 4.9 × 10^–4^ at P_50_ and P_95_, respectively. These equate to nearly one chance in 100,000 people and five chances in 10,000, respectively. The estimated cancer risk values of HCH isomers were also higher than 10^–6^. For example, at P_50_ concentration, khat varieties displayed CR values ranging from 1.57 × 10^–5^ to 2.53 × 10^–5^ for β*-*HCH (Fig. 3b). Whereas at P_95_, CR increased only to 2.61 × 10^–5^–4.08 × 10^–5^, which means khat consumers in South Wollo would have approximately two to four chances in 100,000 for getting cancer from a life time exposure of β*-*HCH. Estimates of risk associated with khat consumption is different in different areas depending upon the level of contamination and chewing pattern. In comparison, high-end consumption probability would have promoted five times higher OCP-related health risks (non-cancer and cancer risks).

Overall, the results of the present study could contribute to the management of OCPs, not only in South Wollo but also in other areas of the country with similar khat cultivation practices. Because khat is chewed fresh without being subjected to washing, drying and heat treatments, certainties associated with estimated intakes and health risk should be high. There are, however, a number of limitations in this study. First, different risks associated with separate age and gender groups were not considered. Second, no consumption survey was conducted to estimate the frequency and amount of khat consumption by the locals in the sub-region. Developing guidelines that achieve a balance between promoting health and protecting other benefits, including farmer and fishermen livelihoods and a safe supply, is challenging and depends on adequate health data. The multiple cumulative DDT and HCH exposure is affecting the population in Africa through OCP contamination in fish and other animal-based foods (Yohannes et al. 2014; Gerber et al. 2016; Pheiffer et al. 2018). This exposure must be comprehensively examined to prioritize risk reduction steps and to target vulnerable groups.

## Conclusion

In the present study, 100% of the khat samples contained *p*,*p*′*-*DDT and β*-*HCH, with significant differences among khat varieties for the former. Despite similar frequency of occurrence of *p*,*p*′-DDT and β*-*HCH, *p*,*p*′*-*DDT median concentrations were lower than β*-*HCH except for Kemissie-Normal, which is probably one of the most contaminated khat type in terms of DDT. One hundred percent of the khat samples contained β*-*HCH above the MRL set by the EC, whereas for total DDT this was the case for 25.7% of the samples. From chemical signatures, we concluded that DDT was freshly applied in khat-based agroecosystems. Still, khat growers may prefer illicit DDT use because they can generate far higher earnings than with legitimate cultivation. Thus, illicit accessibility of DDT must be curtailed through a proper national pesticide control scheme. On the other hand, the very low detection frequency of α-HCH and the high concentration values for β*-*HCH indicate HCH residues from historical usage. For both *p*,*p*′*-*DDT and β*-*HCH, the health risk values were found to be far lower than one, indicating no obvious non-cancer risk. Nevertheless, consumption of khat surpassed the cancer risk limits for total DDT, which shall warrant concern. In future, when assessing the per capita intakes of OCPs, we should not only consider khat and vegetables, but also monitor the contamination levels in staple food crops and animal-based foods and associated health risks. Importantly, assessing gender-related OCP exposure using human hair as a biomarker may expand knowledge about public health disorders. Furthermore, efforts must be continued to build a pesticide database in the country.

## Supplementary Information

Below is the link to the electronic supplementary material.

Supplementary file1 (DOCX 22 kb)

## Data Availability

Not applicable.
