# Supplementary material for: Exposure to DDT and HCH congeners and associated potential health risks through khat (Catha edulis) consumption among adults in South Wollo, Ethiopia
Source: Environ Geochem Health. 2021 Feb 17;43(9):3597–613. doi: 10.1007/s10653-021-00846-w (PMC7886647; doi:10.1007/s10653-021-00846-w)
Supplement: Supplementary file 1 — Supplementary file1 (DOCX 22 kb) [file 10653_2021_846_MOESM1_ESM.docx]

Table S1: Composition of the internal standard mix used (stock solution). Sorted after concentration.

| Component | Concentration [µg mL^-1^] |
| --- | --- |
| *p.p’*-DDE-D8 | 5 |
| *p.p’*-DDD-D8 | 10 |
| ^13^C-*o.p’*-DDT | 15 |
| ^13^C-*p.p’*-DDT | 15 |
| *α*-HCH-D6 | 15 |

Table S2: Retention times and ions used to identify and quantify the analytes. RT = Retention time, IS = Internal standard.

| Name | Purpose | RT [min] | Quantifier [m/z] | Qualifier [m/z] |
| --- | --- | --- | --- | --- |
| *α*-HCH-D6 | IS | 21.147 | 224 | 185 |
| *α*-HCH | Analyte | 21.362 | 219 | 181 |
| *γ*-HCH | Analyte | 22.703 | 219 | 181 |
| *β*-HCH | Analyte | 23.919 | 219 | 181 |
| *δ*-HCH | Analyte | 24.980 | 219 | 181 |
| *o.p’*-DDE | Analyte | 29.020 | 246 | 318 |
| *p.p’*-DDE-D8 | IS | 30.370 | 254.2 | 326 |
| *p.p’*-DDE | Analyte | 30.460 | 246 | 318 |
| *o.p’*-DDD | Analyte | 30.820 | 235 | 165 |
| ^13^C-*o.p’*-DDT | IS | 32.000 | 247 | 177 |
| *o.p’*-DDT | Analyte | 32.008 | 235 | 165 |
| *p.p’*-DDD-D8 | IS | 32.379 | 243 | 173 |
| *p.p’*-DDD | Analyte | 32.500 | 235 | 165 |
| ^13^C-*p.p’*-DDT | IS | 33.682 | 247 | 177 |
| *p.p’*-DDT | Analyte | 33.683 | 235 | 165 |

Table S3: Results of the method validation. LOD = Limit of detection, LOQ = Limit of quantification, RSD = Relative standard deviation. n = 5 for DDX and n = 4 for HCH.

|  | DDX | | | | | | HCH | | | |
| --- | --- | --- | --- | --- | --- | --- | --- | --- | --- | --- |
|  | *p*,*p*’-DDT | *o*,*p*’-DDT | *p*,*p*’-DDD | *o*,*p*’-DDD | *p*,*p*’-DDE | *o*,*p*’-DDE | *α* | *β* | *γ* | *δ* |
| LOD [µg/kg] | 0.1 | 0.1 | 0.1 | 0.1 | 0.1 | 0.1 | 0.3 | 0.15 | 0.45 | 0.3 |
| LOQ [µg/kg] | 0.3 | 0.3 | 0.3 | 0.3 | 0.3 | 0.3 | 1 | 0.5 | 1.5 | 1 |
| Recovery rate [%] | 86.72 | 86.72 | 95.75 | 114.75 | 89.10 | 89.60 | 107.74 | 97.33 | 111.12 | 103.13 |
| RSD [%] | 8.84 | 6.14 | 8.73 | 10.70 | 6.39 | 4.87 | 2.84 | 11.74 | 5.51 | 10.12 |
